# Impact of NMDA Receptor Overexpression on Cerebellar Purkinje Cell Activity and Motor Learning

**DOI:** 10.1523/ENEURO.0270-17.2018

**Published:** 2018-02-12

**Authors:** Elisa Galliano, Martijn Schonewille, Saša Peter, Mandy Rutteman, Simone Houtman, Dick Jaarsma, Freek E. Hoebeek, Chris I. De Zeeuw

**Affiliations:** 1 Department of Neuroscience, Erasmus Medical Centre, Rotterdam, The Netherlands; 2 Netherlands Institute for Neuroscience, Royal Netherlands Academy of Arts and Sciences, Amsterdam, The Netherlands

**Keywords:** cerebellum, compensatory eye movements, motor learning, NMDA, Purkinkje cell, synaptic plasticity

## Abstract

In many brain regions involved in learning NMDA receptors (NMDARs) act as coincidence detectors of pre- and postsynaptic activity, mediating Hebbian plasticity. Intriguingly, the parallel fiber (PF) to Purkinje cell (PC) input in the cerebellar cortex, which is critical for procedural learning, shows virtually no postsynaptic NMDARs. Why is this? Here, we address this question by generating and testing independent transgenic lines that overexpress NMDAR containing the type 2B subunit (NR2B) specifically in PCs. PCs of the mice that show larger NMDA-mediated currents than controls at their PF input suffer from a blockage of long-term potentiation (LTP) at their PF-PC synapses, while long-term depression (LTD) and baseline transmission are unaffected. Moreover, introducing NMDA-mediated currents affects cerebellar learning in that phase-reversal of the vestibulo-ocular reflex (VOR) is impaired. Our results suggest that under physiological circumstances PC spines lack NMDARs postsynaptically at their PF input so as to allow LTP to contribute to motor learning.

## Significance Statement

NMDA receptors (NMDARs) form one of the key molecules involved in memory formation, yet they are absent in one of the most plastic synapses in the brain, the parallel fiber (PF) to Purkinje cell (PC) synapse. PCs are equipped with the molecular machinery for expression of NMDARs, but under normal conditions all NMDARs are directed toward their climbing fiber (CF) input. So why are NMDARs not occurring postsynaptically at their PF input, which is known as the main substrate of cerebellar plasticity? To answer this question, we generated two transgenic mouse lines that overexpress NMDARs at the PF-PC synapse, and we show that while such manipulation does not result in abnormal morphology or baseline PC electrophysiology, it does impact plasticity and motor learning.

## Introduction

A basic property of chemical synapses is their ability to be permanently modified in response to stimulus patterns. This property, which is known as long-term synaptic plasticity, can result in potentiation or depression of transmission, and is thought to serve as a cellular basis for memory formation (Bliss and Lomo, 1973). The existence of long-term synaptic plasticity was predicted by Donald Hebb, who postulated that the synaptic connection between two neurons would be strengthened when pre- and postsynaptic elements are active simultaneously (Hebb, 1949). Such a process implies the existence of a coincidence detector able to sense at the same time the pre- and postsynaptic activity. The NMDA receptor (NMDAR), which is a cationic channel sensitive for glutamate released at the presynaptic site and voltage changes at the postsynaptic site, is a well-known coincidence detector, the activity of which appears to be key to the induction of various types of synaptic plasticity in the CNS (Cotman et al., 1988). However, in the cerebellar Purkinje cell (PC), a neuron with great capacity for synaptic plasticity (Carey, 2011; Gao et al., 2012), things are peculiar. First, NMDARs in PCs are expressed relatively late, after the second postnatal week; second, their NMDARs are expressed at a relatively low level; and finally, NMDAR currents in PCs can only be measured at just one of their inputs, i.e., the climbing fiber (CF) synapse (Piochon et al., 2007; Renzi et al., 2007), where they are required for the induction of long-term depression (LTD) at the parallel fiber (PF) to PC synapse (PF-PC LTD; Piochon et al., 2010). So why are there virtually no functional NMDARs postsynaptically at the other glutamatergic afferent to PCs, i.e., the PF synapse? This is an intriguing question, especially given the facts that PF-PC synapses are abundant (between 100,000 and 200,000 for each PC), that these synapses show multiple forms of plasticity, and that they even express NMDARs presynaptically (Bidoret et al., 2009; Bouvier et al., 2016). One could speculate that since the opening of NMDARs will induce a calcium influx (Nicoll and Malenka, 1995), and the level of calcium influx in turn will determine to what extent a PF-PC synapse will be potentiated or depressed at its postsynaptic site (Coesmans et al., 2004), it is possible that the virtual absence of NMDARs at this synapse is necessary to keep the local calcium transients at PC spines relatively low and thereby allow long-term potentiation (LTP) to occur. Here, we set out to test the hypothesis that the lack of NMDARs at the PF-PC synapse is permissive for LTP induction by generating novel PC specific transgenic mouse lines that overexpress the NR2B subunit of NMDARs under the L7-promotor (L7-NR2B+/Tg1 and L7-NR2B+/Tg2; hereafter referred to as Tg1 and Tg2) and by subsequently investigating their cellular and behavioral consequences. We selected the form B of the NR2 subunit, because this is the most permeable subunit for Ca^2+^, it has been shown to enhance synaptic plasticity, it is not expressed by any other neuron in the cerebellar cortex, and it has previously been inserted successfully in neuronal cultures, forming functional units with NR1 subunits (Monyer et al., 1994; Feldmeyer and Cull-Candy, 1996; Kakegawa et al., 2003). To minimize the possibility that all new overexpressed NMDARs would be directed to the CF synapse as occurs under physiologic circumstances (Piochon et al., 2007; Renzi et al., 2007), we overexpressed NR2B subunits with truncated UTR sites, which might serve as regulatory elements for subcellular trafficking (Wood et al., 1996; Di Liegro et al., 2014). Our data show that such overexpression of NR2B subunits in PCs *in vivo* results in functional NMDARs and that many of the genetically induced, additional NMDA-mediated currents are located at the PF-PC synapse. Moreover, overexpression of NR2B impaired induction of LTP, but not LTD, at the PF-PC synapse, and affected adaptation of the vestibulo-ocular reflex (VOR), which is known to depend on LTP (Schonewille et al., 2010; De Zeeuw and Ten Brinke, 2015; Gutierrez-Castellanos et al., 2017; Voges et al., 2017). Together, these findings highlight the quintessence of regulating the low and site-specific expression of NMDAR subunits in PCs, setting it apart from that in other neurons involved in memory formation (Kessels et al., 2013; Nabavi et al., 2013).

## Materials and Methods

### Generation of transgenic mice

The NR2B transgene (Mouse Grin2b-001; ENSMUST00000053880.12) including 470 bases of the 3’ UTR, was cloned into expression vector pGEM-L7containing the L7-promoter (Barski et al., 2000), by fusing the ATG initiation codon of the NR2B transgene with the initiation codon of the L7 gene. The resulting pGEM-L7NR2B was digested with with SalI/ClaI and the insert was used to generate the transgenic founders by pronuclear injection into C57BL/6NHsd inbred zygotes. The inbred founders were crossed into C57BL/6NHsd to produce F1 generation, two of which were selected for F2 offspring (Tg1 and Tg2) against a C57BL/6NHsd background. The genotypes of all offspring were analyzed by preparing tail DNAs. The 5' and 3' primers for detecting NR2B transgene were L7 S (CAC TTC TGA CTT GCA CTT TCC TTG G), L7 AS (TTC TTC AAG CTG CCC AGC AGA GCT C) and 165 (GCC AAA CTG GAA GAA CAT GGA GGA C); wild-type 450 bp, transgenic 557 bp. Mouse tail DNAs (∼1 mg) were amplified (94°C 3'//94°C 1’; 58°C 45''; 72°C 1':30 cycles//72°C 10'//4°C) on a Robo cycler. For all experiments the researchers were blind to the genotype of the animals. Unless stated otherwise we used 6 ± 1-week-old male and female littermates, gender-matched across groups. All experiments were performed in accordance with the guidelines for animal experiments of the Erasmus MC, Netherlands Institute for Neuroscience (KNAW), and the Dutch National Legislation.

### Western blotting

Lysates for Western blotting were prepared by quick dissection of the brain and by homogenization of brain tissue in lysis buffer (10 mM TRISHCl 6.8, 2.5% SDS, 2 mM EDTA) and protease and phosphatase inhibitor cocktails (Sigma). The concentration of the lysates was adjusted to 1 mg/ml and a 10-μg volume and was used for Western blot analysis. Western blottings were probed with antibodies directed against the N-terminal NR2B (anti-mouse, 1:1000; Cell Signaling) and NR1 (anti-rabbit, 1:1000; Cell Signaling). Bands were visualized using Enhanced Chemo Luminescence (Pierce). Loading controls were performed with anti-actin antibodies (1:20,000, Cell Signaling).

### Histology

Mice were anesthetized with an overdose of Nembutal (intraperitoneal) and transcardially perfused with saline followed by 4% paraformaldehyde (in 0.12 M phosphate buffer; PB). Sagittal sections (40 μm thick) were processed free-floating for calbindin immunohistochemisty or NR2B-immunofluoresence. For NR2B-immunofluoresence sections were exposed to limited proteolytic digestion to expose synaptic receptors (Watanabe et al., 1998): Sections were incubated in 0.2 M HCl containing 0.4 mg/ml pepsin (Sigma) for 20 min with continuous agitation. After rinsing in PBS and preincubation with PBS with 0.1% Triton X-100 (PBST) and 10% normal horse serum, the sections were incubated in PBST/1% normal horse serum with an anti-NR2B antibody (NeuroMab clone N59/36; dilution 1:1000) for 48 h at 4°C. NR2B antibody was visualized with Cy3-donkey anti-mouse secondary antibody (1:400), and analyzed with an LSM 700 upright confocal laser scanning microscope (Carl Zeiss, Jena, Germany). For calbindin immunohistochemistry, after preincubation in PBST sections were incubated with rabbit anti-calbindin antibody (Swant; dilution 1:15000) in PBST/1% normal horse serum for 48 h at 4°C, followed by incubation with biotinylated goat-anti-rabbit secondary antibody (1:200; Vector), incubation with avidin-biotin-peroxidase complex (ABC; Vector Laboratories), and reaction with diaminobenzidine (DAB, 0.05%). Calbindin-immunoperoxidase-stained sections were analyzed using a Leica DM-RB microscope, or scanned with a Hamamatsu NanoZoomer 2 whole slide imager and analyzed with NDP.view (Hamamatsu City) software. For Golgi staining, which was used for Sholl analysis, Tg1-, Tg2-, and control mice were perfused with saline, followed by a buffered aldehyde fixative and a mordant consisting of 6% potassium dichromate, 6% chloral hydrate, and 4% formaldehyde. After postfixation in the same mordant for 3 d, blocks of the cerebellar cortex were treated with 0.75% silver nitrate for an additional 3 d, embedded in a soft Epon mixture and sectioned with a heated steel knife (De Zeeuw et al., 1996).

### *In vitro* electrophysiology

Mice (6 ± 1 weeks old) were decapitated under isoflurane anesthesia. Subsequently, the cerebellum was removed and transferred into ice-cold slicing medium containing: 240 mM sucrose, 5 mM KCl, 1.25 mM Na_2_HPO_4,_ 2 mM MgSO_4_, 1 mM CaCl_2_, 26 mM NaHCO_3_, and 10 mM D-glucose, bubbled with 95% O_2_ and 5% CO_2_. Parasagittal slices (200 or 250 μm thick) of the cerebellar vermis were cut using a vibratome (VT1000S, Leica) and afterward kept in ACSF containing: 124 mM NaCl, 5 mM KCl, 1.25 mM Na_2_HPO_4_, 2 mM MgSO_4_, 2 mM CaCl_2_, 26 mM NaHCO_3_, and 20 mM D-glucose, bubbled with 95% O_2_ and 5% CO_2_ for > 1 h before the experiments started. *In vitro* experiments were performed in slices at room temperature (20–22°C), which were continuously perfused with ACSF that was supplemented with picrotoxin (100 μM) or bicuculline methiodide (20 μM) to block GABA_A_ receptors. All drugs were purchased from Sigma. Whole-cell patch-clamp recordings were performed using either an EPC-10 amplifier (HEKA Electronics) or an Axopatch amplifier 700B (Molecular Devices). PCs were visualized using an upright microscope (Axioskop 2 FS plus, Carl Zeiss) equipped with a 40× water immersed objective. If not stated otherwise, recording electrodes were filled with an intracellular solution containing: 124 mM K-gluconate, 9 mM KCl, 10 mM KOH, 4 mM NaCl, 10 mM HEPES, 28.5 mM sucrose, 4 mM Na_2_ATP, and 0.4 mM Na_3_GTP (pH 7.25–7.35; osmolarity ∼290). For extracellular stimulation, patch electrodes filled with ACSF were positioned to touch the surface of the slice at the most distal 1/3 of the molecular layer lateral to the recorded PCs for PF stimulation and in the granular layer close to the recorded PC for CF stimulation. Recordings were excluded if series or input resistances (R_S_ and R_I_, respectively; assessed by -10 mV voltage steps following each test pulse) varied by >15% over the course of the experiment. The liquid junction potential was not corrected for. Quantification of NMDA currents was performed in magnesium-free ACSF supplemented with glycine (10 µM, Sigma) by subsequent application of the AMPA antagonist NBQX (12.5 µM, Tocris) and the NMDA antagonist D-AP5 (10 µM, Tocris). All-or-none CFs responses were first identified in current clamp by the typical complex spike shape and the absolute NMDA contribution was calculated in voltage clamp (holding potential -65 mV) by subtracting the NBQX + AP5 insensitive residual component to the NBQX sensitive. PF responses of approximately -200 pA were taken as a baseline in voltage clamp (holding potential -65 mV) and the NMDA contribution was calculated as described for CF responses but normalized to the baseline response. Rs was compensated online to obtain a residual value >10 MΩ. PC intrinsic excitability was recorded in current-clamp mode at 34 ± 1°C. PCs were discarded when >800 pA hyperpolarizing current was required to maintain the holding potential at -65 mV or when action potential firing occurred at this holding potential. We injected 800-ms current steps ranging from -100 to +1000 pA with 100-pA increments. The average spiking frequency measured over the entire current pulse was used to construct current-frequency plots. Action potential properties (peak amplitude, after-hyperpolarization amplitude and half-width) were evaluated using the first action potential generated by each PC. To assess the stimulus intensity, EPSC amplitude (input-output) ratio consistently, only PCs were selected in regions with comparable width of the molecular layer. For these recordings, electrodes contained: 130 mM CsMeSO_4_, 4 mM MgCl_2_, 0.2 mM EGTA, 10 mM HEPES, 10 mM Na-phosphocreatine, 1 mM QX-314, 4 mM Na_2_ATP, and 0.4 mM Na_3_GTP (pH 7.25–7.35). LTP at the PF-PC synapse was assessed by PF stimulation at 1 Hz for 5 min (PF-LTP protocol; Coesmans et al., 2004), while LTD was induced by PF activation (10 stimuli at 100 Hz) and CF activation (2 stimuli at 20 Hz), repeated 30 times every 10 s at 34 ± 1°C (PF-LTD protocol; Safo and Regehr, 2005). Test responses were evoked at a frequency of 0.05 Hz (two stimuli of 0.5–6 μA pulses; 50-ms interstimulus interval). PCs were clamped at -65 to -70 mV to prevent spontaneous action potential firing. Plasticity of PC’s intrinsic excitability was recorded in current-clamp mode at 34 ± 1°C. PCs were discarded when >800 pA hyperpolarizing current was required to maintain the holding potential at -65 mV or when action potential firing occurred at this holding potential. We induced intrinsic plasticity (IP) by 1 Hz PF stimulation for 5 min (PF-LTP protocol, comparable to Belmeguenai et al., 2010, but with short ramping current injections to probe the response; Fig. 3*D*) at I = 0. Current steps (800 ms), ranging from 100 to 800 pA in 100 pA increments were injected to evoke action potential firing during steps 2–4. The spike count at the third current step was taken as a measure of excitability. Ri was calculated from the first hyperpolarizing current injection. To determine whether plasticity was induced a linear mixed model was used based on dividing the post-tetanus period into two 15-min periods (post1 and post2) and comparing these to pre-tetanus values (pre). For LTP, LTD, and IP the estimates of fixed effects (of tetanus stimulus) on EPSCs in control mice were: LTP, post1 vs pre, estimated +25.9 ± 4.9%, *p* < 0.001; post2 vs pre, estimated +21.3 ± 5.0%, *p* < 0.001; LTD, post1 vs pre, estimated –21.7 ± 5.5%, *p* < 0.001; post2 vs pre, estimated –12.8 ± 5.6%, *p* = 0.022; LTP with D-AP5, post1 vs pre, estimated +10.2 ± 1.9%, *p* < 0.001; post2 vs pre, estimated +6.9 ± 1.9%, *p* < 0.001; and IP, post1 vs pre, estimated –4.3 ± 4.5%, *p* =0.34; and post2 vs pre, estimated +16.1 ± 4.6%, *p* = 0.001. To compare genotypes a repeated measures ANOVA was used on the 20-min recording period post tetanus (the minimal recording duration).

### Open field

To test locomotor activity individual mice were placed in a circular, dimly-lit open field (120 cm in diameter), and the total distance traveled, together with the average speed of each mouse, was recorded for 10 min (SMART software, Panlab).

### Compensatory eye movements

During their fifth postnatal week mice were prepared for chronic, head-restrained recordings of compensatory eye movements. Mice were 37 ± 3 d at the beginning of the 5 d of compensatory eye movement testing. In short, under isoflurane anesthesia (initiation at 4%, maintenance at ∼1.5% with O_2_) a pedestal was constructed using Optibond primer and adhesive (Kerr) and Charisma (Haeraeus Kulzer). After a recovery period (2–3 d) mice were head-fixed by means of a magnet (Neodymium, 4 × 4 × 2 mm, MTG Europe) embedded in a custom-made U-shaped pedestal and a securing screw. A cylindrical screen (diameter 63 cm) with a random-dotted pattern (each element 2°) surrounded the turntable (diameter 60 cm) on which the mouse was placed. The optokinetic reflex (OKR) and the VOR in dark and VOR in light (visually-enhanced VOR; VVOR) were elicited by sinusoidal rotation of either drum (OKR) or table (VOR and VVOR). Motor performance was tested by rotating at 0.1–1.0 Hz with 5° amplitude (fixed). Each frequency-amplitude combination was tested twice with 8 (for 0.1 Hz) to 20 (for 1.0 Hz) repeated cycles and results were averaged. Motor learning was tested by mismatching visual and vestibular input: “gain decrease” was evoked by rotating drum and table in phase at the same amplitude (5°) at 0.6 Hz and “phase reversal” by subsequent rotation in phase with increased amplitude of the drum (day 2, 7.5°; day 3, 10°) at the same frequency. Animals were kept in the dark in between training sessions. Phase reversal results are depicted as gain multiplied by the cosine of the phase to capture the change in timing and amplitude of movement in a single value (gain*cos(phase)). Phase values larger than 90° result in a negative gain*cos(phase) value. Consolidation was calculated as the learned response on the second day as a percentage of the learning during the first day; for example, gain decrease consolidation = 100% · (g_max-day1_ – g_max-day2_/(g_max-day1_ – g_min-day1_), with g_min-day1_ being the minimum gain on day 1 and g_max-day2_ the maximum gain on day 2. To illuminate the eye during the recordings we used two table-fixed infrared emitters (OD-50L, maximum output 600 mW, dispersion angle 7°, peak wavelength 880 nm; Opto-Diode), and a third emitter mounted to the camera aligned horizontally with the camera’s optical axis. This third emitter produced the tracked corneal reflection. The pupil position, after subtraction of the corneal reflecting position, was recorded using the eye-tracking device (ETL-200, ISCAN Systems). Calibrations were performed as described previously (Stahl et al., 2000). Gain and phase values of the eye movements were calculated using a custom-made Matlab routine (Matlab, MathWorks Inc). Gain and phase results plotted against frequency or time were statistically analyzed using repeated measures ANOVA.

### Data analysis

All values are represented as mean ± SEM, *p* values of <0.05 were considered significant. Data distributions were evaluated using either Levene’s test of equality of variance for independent data or Mauchly’s test of sphericity for repeated measures. Unless stated otherwise, statistical analysis was done using one-way ANOVA test with Tukey’s *post hoc* correction where three groups were compared (for details per test, see Table 1).

**Table 1.** Statistical testing

Figure	Data	Data structure	Type of test	Actual power
1*E*	Intersections w/Sholl circles	Normal distr.	Repeated measures ANOVA	0.236
1*E*	Primary dendrite length	Normal distr.	One-way ANOVA	0.944
2*A*	CF-PC NMDA current	Normal distr.	One-way ANOVA	0.988
2*B*	PF-PC NMDA current	Non-normal distr.	Kruskal–Wallis test	0.940
2*C*	I-O Linear fit slope	Normal distr.	One-way ANOVA	0.295
	I-O First spike peak amplitude	Normal distr.	One-way ANOVA	0.369
	I-O First spike AHP amplitude	Normal distr.	One-way ANOVA	0.769
	I-O First spike half-width	Normal distr.	One-way ANOVA	0.319
2*D*	slope EPSC I-O	Normal distr.	Repeated measures ANOVA	0.077
3*A*	PF-PC LTP group comparison	Normal distr.	Repeated measures ANOVA	0.592
3*B*	PF-PC LTD group comparison	Normal distr.	Repeated measures ANOVA	0.079
3*C*	PF-PC LTP with D-AP5, groups	Normal distr.	Repeated measures ANOVA	0.057
3*D*	IP, 10 min	Normal distr.	Repeated measures ANOVA	0.589
3*D*	IP, 20 min	Normal distr.	Repeated measures ANOVA	0.386
4*A*	open field, path length	Normal distr.	Two-sample *t* test	0.283
	open field, average speed	Normal distr.	Two-sample *t* test	0.280
4*B*	OKR, gain	Normal distr.	Repeated measures ANOVA	0.071
	OKR, phase	Normal distr.	Repeated measures ANOVA	0.086
4*C*	VOR, gain	Normal distr.	Repeated measures ANOVA	0.136
	VOR, phase	Normal distr.	Repeated measures ANOVA	0.314
4*D*	VVOR, gain	Normal distr.	Repeated measures ANOVA	0.191
	VVOR, phase	Normal distr.	Repeated measures ANOVA	0.105
4*E*	VOR gain decrease training	Normal distr.	Repeated measures ANOVA	0.157
	VOR gain decr consolidation	Normal distr.	Two-sample *t* test	0.329
4*F*	VOR gain*phase reversal day 2	Normal distr.	Repeated measures ANOVA	0.050
	VOR gain*phase reversal day 3	Normal distr.	Repeated measures ANOVA	0.862
	VOR gain*phase reversal day 4	Normal distr.	Repeated measures ANOVA	0.635
	VOR gain*phase reversal day 5	Normal distr.	Repeated measures ANOVA	0.948

## Results

### Transgenic overexpression of NR2B in PCs increases CF-PC NMDA responses and introduces functional NMDARs in PF-PC synapses

**Figure 1.**
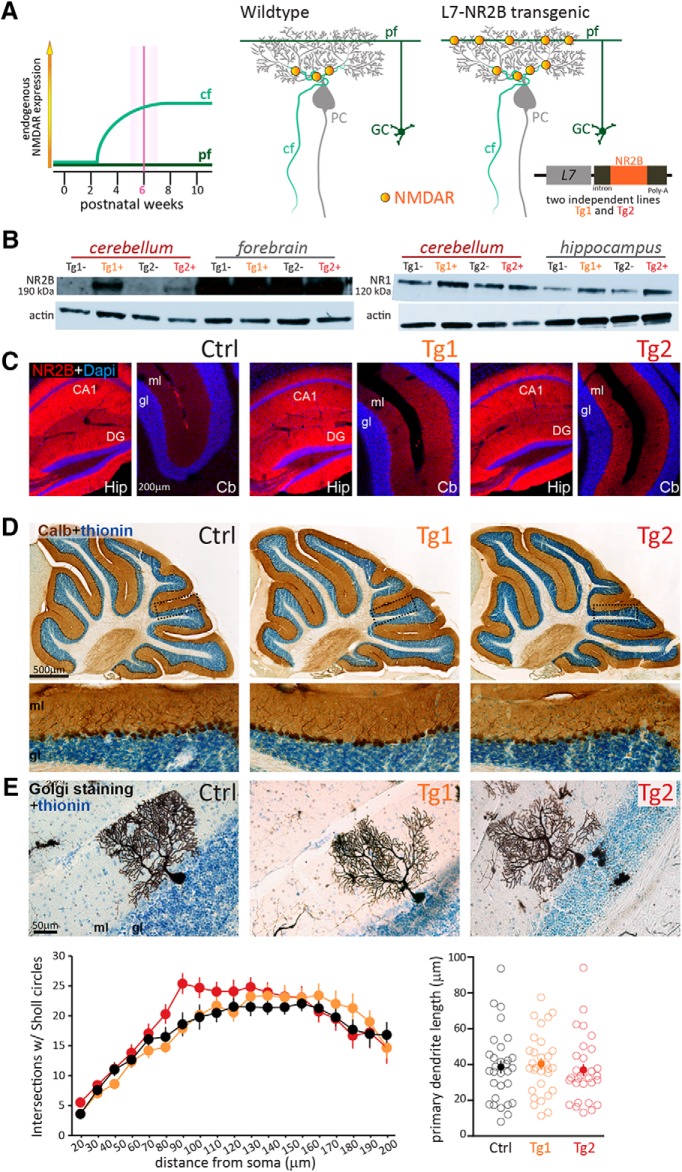
Generation of NR2B transgenic mice. ***A***, Schematic representation of the expression of endogenous NMDARs at CF-PC and PF-synapses over the wild-type mouse lifetime. All experiments performed in the following figures are performed at 6 ± 1 weeks of age (for details, see Materials and Methods). Middle, Scheme depicting the expression of NMDAR (dots) in a wild-type mouse at the synapses formed on the PC dendritic tree by CFs. Synapses formed by PFs (the axons of granule cells, GC) do not have NMDARs. Right, Same schematic representation of synapses onto PCs in the L7-NR2B + Tg transgenic mouse. Note that NMDARs are present also at the PF-PC synapse. Inset, Details of the vector used to generate the two independent lines used in the study. ***B***, Western blotting gels containing homogenates of adult cerebella, forebrains, and hippocampi of two transgenic mice (Tg1+ and Tg2+) and their control littermates (Tg1- and Tg2-). The left blot was processed with an anti-NR2B antibody, which visualizes a band at 190 kDa, the right one with an anti-NR1 antibody (120 kDa). Actin was used as loading control; note that the ratio of NR labeling to actin labeling should be taken into account for assessing the amounts of protein present. ***C***, Confocal immunofluorescent images of NR2B-immunoreactivityin dorsal hippocampus (Hip) and cerebellar cortex (Cb) in control (left), Tg1 (middle), and Tg2 (right) mice. Note in control mice, the low level of NR2B labeling (blue) in the cerebellum as compared to hippocampal CA1 and dentate gyrus (DG). In addition, note moderate increased labeling in cerebellar molecular layer (ml) of transgenic mice. ***D***, Low- and high-magnification images of calbindin immunoreactivity in sagittal cerebellar sections illustrating the normal appearance of cerebellar gross morphology and PCs of adult transgenic (Tg1 and Tg2) mice. ***E***, top, High magnification of individual Golgi-stained PCs (black) of control (left) as well as Tg1 (middle) and Tg2 (right) NR2B mice, counterstained with thionin (blue). Bottom, Sholl analysis of the dendritic arborization of PCs (left) and length of their primary dendrites (right) for control (black, *n* = 30, *N* = 4), Tg1 (orange, *n* = 30, *N* = 3), and Tg2 (red, *n* = 30, *N* = 4) NR2B mice. Empty circles indicate individual data points, full circles indicate mean ± SEM.

**Figure 2.**
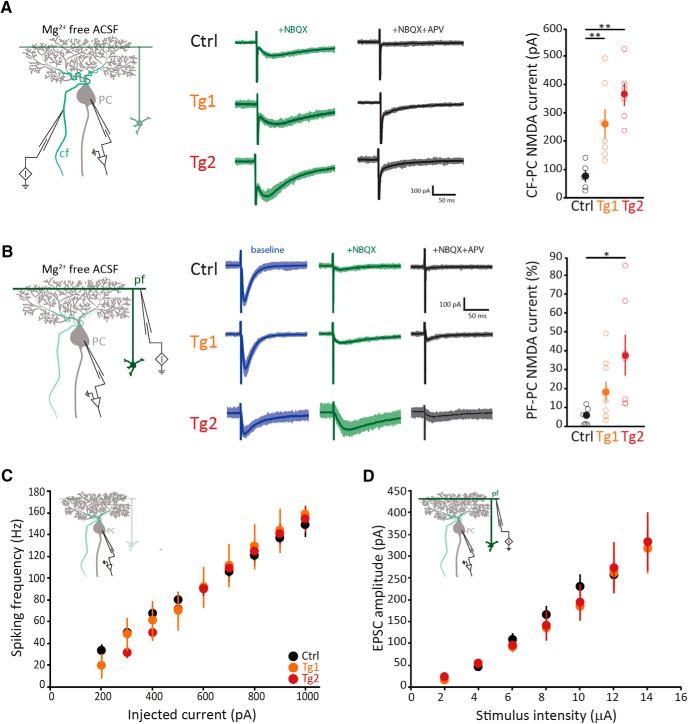
Functional NMDARs are present at six weeks of age and do not compromise PCs’ basic electrophysiological properties. ***A***, left, Schematic representation of the recording configuration. Middle, Example traces of CF currents recorded in the presence of the AMPA antagonist NBQX (green) and subsequently of blockers of both AMPA and NMDARs (D-AP5, black) in PCs of both transgenic lines and control littermates. Bold lines are average values; shading indicates individual cell variability. Right, Quantification of NMDA current at the CF-PC synapse in control (Ctrl, black, *n* = 5) and transgenic animals (Tg1, orange, *n* = 7; Tg2, red, *n* = 6). ***B***, Similar to ***A***, with additional example traces of baseline PF-evoked EPSCs before the addition of glutamatergic receptors blockers (blue), and normalized PF-PC NMDA current quantification (Ctrl, *n* = 9; Tg1, *n* = 9; Tg2, *n* = 7). Note that NMDA-mediated currents are only significantly different from controls in Tg2. ***C***, Average firing frequency elicited by somatic current injections from -65 mV in PCs of transgenic (Tg1, orange, *n* = 5; Tg2, red, *n* = 12) and control (Ctrl, black, *n* = 15) mice. The inset illustrates the recording configuration. ***D***, Average amplitude of the EPSCs at the PF-PC synapse to stimuli of increasing intensity for transgenic (Tg1, orange, *n* = 5; Tg2, red, *n* = 8) and control (Ctrl, black, *n* = 18) mice. The inset illustrates the recording configuration**. **Empty circles represent individual data points, full circles are mean ± SEM; *p < 0.05, **p < 0.01, and absolute *p* values are indicated in the main text.

NMDARs are complex, heterotetrameric channels formed by two NR1 subunits and two NR2 subunits (Tovar and Westbrook, 2017). Under normal developmental and physiologic circumstances NR1 is expressed by PCs directly after birth, while NR2 subunits, required to form functional receptors at the CF-PC synapses, are not expressed until two to three weeks after birth, reaching a plateau at approximately two months (Piochon et al., 2007; Fig. 1*A*). To introduce functional NR1/NR2 NMDA currents at the PF-PC synapse, we generated transgenic mouse lines overexpressing the NR2B subunit by inserting the linearized NR2B transgene without its 5'-UTR and most of its 3'-UTR (leaving only 470 bases) under control of the Pcp-2/L7 promoter in an expression vector (De Zeeuw et al., 1998; Barski et al., 2000), which was pronuclearly injected into a C57BL/6 inbred zygote (for detailed procedures, see Materials and Methods). Western blot analysis at six weeks of age confirmed the overexpression of NR2B protein in the cerebellum of two lines of L7-NR2B+ transgenic animals, Tg1 and Tg2 (Fig. 1*B*). In accord with increased expression of NR2B in PCs, immunofluorescence showed increased NR2B staining in the cerebellar molecular layer of both transgenic lines (Fig. 1*C*). Western blotting and immunohistology also indicated that in both transgenic lines labeling in the cerebellum was still considerably lower than that in hippocampus and cortex, which are known for very high levels of NR2B expression (Monyer et al., 1994). The ratio of NR labeling to loading control actin labeling indicated a relatively high amount of the actual protein present in Tg2, but the generally low expression levels and suboptimal antibody quality, commonly seen with channel receptors, prohibited an accurate evaluation of the subcellular localization or quantification of the expression. We therefore took these results as qualitative evidence for the presence of NR2B, while the functional consequences and related quantifications were tested with cell physiologic approaches (see Figure 2).

Both Tg1 and Tg2 mice showed normal growth, body weights and breeding ratios compared to control littermates. The cyto-architecture of the cerebellum of both lines was normal and their foliation was well preserved (Fig. 1*D*). Immunohistochemistry for calbindin-D28K and Sholl analysis of Golgi stained tissue indicated that morphology of PCs was unaltered (Sholl analysis: *p* = 0.90 and *p* = 0.32 for Tg1 and Tg2 vs Ctrls, respectively, Rep. measures ANOVA; Primary dendrite length: Tg1 40.3 ± 3.1 µm, Tg2 37.0 ± 3.3 µm, Ctrl 38.6 ± 3.6 µm; *p* = 0.93 and *p* = 0.94 for Tg1 and Tg2 vs Ctrls, respectively) (Fig. 1*E*). Furthermore, behavior of both Tg1 and Tg2 in the home cage was indistinguishable from that of wild-type littermates.

To determine whether ectopic NR2B subunits assemble and result in altered synaptic NMDA currents in PCs, we performed patch-clamp recordings of PCs in acute cerebellar slices bathed in Mg^2+^-free solution with subsequent application of AMPAR and NMDAR blockers (NBQX and D-AP5, respectively). In line with previous studies (Piochon et al., 2007; Renzi et al., 2007), PCs from six-week-old controls showed a NMDA-mediated current (i.e., NBQX-insensitive and AP5-sensitive) at the CF-PC synapse (77 ± 21 pA). In both Tg1 and Tg2 mutant mice the NMDAR-mediated currents at CF-PC synapses were significantly larger than those in controls (262 ± 52 and 365 ± 41 pA; *p* = 0.025 and *p* = 0.001 vs Ctrl, respectively; Fig. 2*A*). Next, we determined whether NMDA-mediated currents also occurred at the PF-PC synapse; i.e., we quantified the percentage of a 200–300 pA PF-PC EPSC that was NMDA mediated. These percentages were negligible in controls (4 ± 1%), modestly present in Tg1 (18 ± 5%), but more prominently present in Tg2 PCs (38 ± 11%); the percentage of Tg2, but not that of Tg1, was significantly higher than that in controls (*p* = 0.001 and *p* = 0.10, respectively; Fig. 2*B*); no obvious difference was observed in the NMDA-mediated EPSC kinetics across groups (all *p* values >0.9).

Next, to study the effect of ectopic NR2B expression on basic PC electrophysiological properties, we first performed whole cell current-clamp recordings at physiologically relevant temperatures to investigate intrinsic excitability (Fig. 2*C*). PCs of all genotypes showed increasing action potential firing frequencies on somatic current injections of increasing amplitude comparable to the ones of their control littermates. The slope of the linear input-output relationship in transgenic mice did not differ from that in control (Ctrls) animals (Tg1 17.4 ± 1.5 Hz/pA, Tg2 18.1 ± 1.9 Hz/pA, Ctrls 15.0 ± 0.9 Hz/pA; *p* = 0.47 and *p* = 0.45 for Tg1 and Tg2 vs Ctrls, respectively), indicating a normal level of excitability. In addition, for each cell we analyzed the action potential properties and again we found no significant differences in terms of spike baseline, peak amplitude, after-hyperpolarization amplitude or half-width among genotypes (all *p* values > 0.13). Finally, the presence of NMDARs in PCs prompted us to investigate the postsynaptic amplitude evoked by PF stimulation (i.e., in a recording solution with Mg^2+^ and holding at -65 mV) at increasing stimulus intensities (Fig. 2*D*). No obvious differences were found in the input/output ratio of PF-EPSCs between transgenic animals and controls (*p* = 0.83, repeated measures ANOVA).

Taken together, our data show that we generated transgenic mice overexpressing NR2B subunits without affecting baseline transmission, but that only Tg2 showed significantly more NMDAR-mediated currents at their PF-PC synaptic inputs compared to Ctrls.

### NMDA currents at the PF-PC synapse selectively prevent the induction of LTP

**Figure 3.**
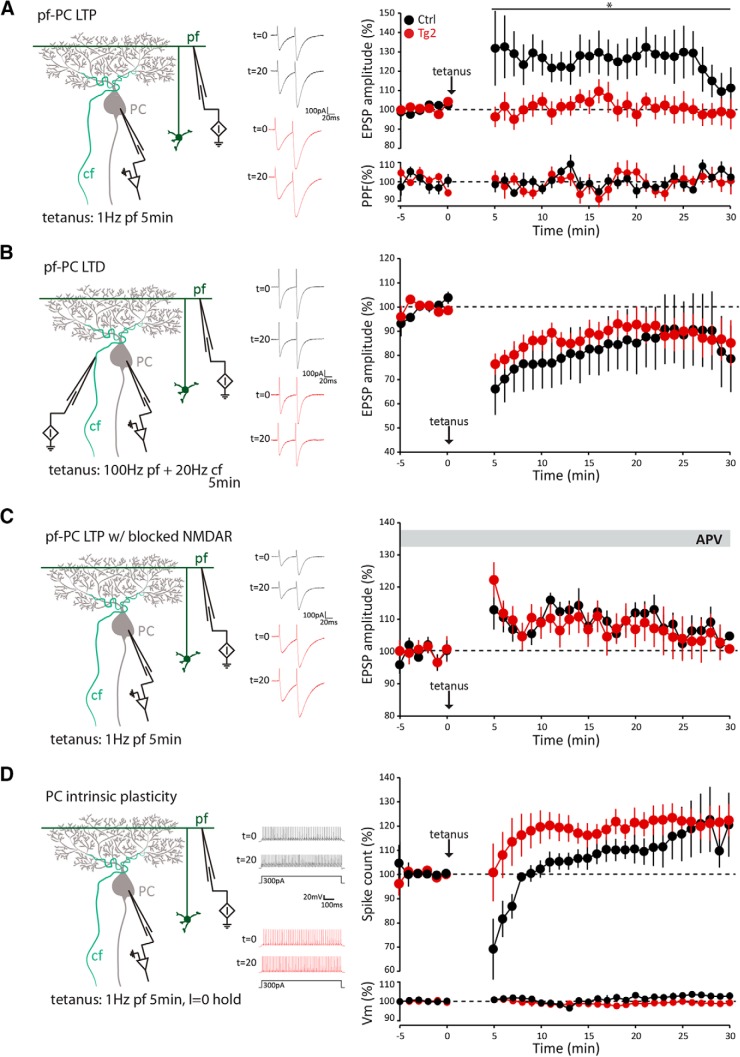
PF-PC LTP is selectively affected in transgenic mice. ***A***, LTP was induced by PF stimulation at 1 Hz for 5 min in six-week-old transgenic (Tg2, red, *n* = 8) and control (Ctrl, black, *n* = 7) mice. The normalized paired-pulse ratio (50-ms interstimulus interval) of the recordings of the same cells is plotted below. ***B***, LTD was induced as described in ***A***, but with concomitant CF activation (Ctrl, *n* = 8; Tg2, *n* = 8). ***C***, Similar to ***A***, but with the NMDAR blocker D-AP5 present in the extracellular solution (Ctrl, *n* = 7; Tg2, *n* = 8). ***D***, Induction of IP by 5 min of PF stimulation at 1 Hz did not result in significant differences between transgenic and control mice (both *N* = 6). The normalized membrane potentials of the same cells are represented below. The scheme at the left of each panel depicts the respective recording configuration, while the middle example traces of Ctrl (black) and Tg2 (red) EPSCs (***A–C***) or action potentials (***D***) recorded before (*t* = 0 min) and after (*t* = 20 min) the tetanic stimuli show the plastic changes. Values are mean ± SEM; *p < 0.05; **p < 0.01; absolute *p* values are indicated in the main text.

While induction of PF-PC LTD depends on CF activation for its high Ca^2+^ influx, LTP is achieved through repetitive stimulation of only PFs and requires a low Ca^2+^ concentration (Coesmans et al., 2004) and has been shown to be independent of postsynaptic NMDARs (Piochon et al., 2010; Wang et al., 2014). We therefore hypothesized that insertion of Ca^2+^-permeable NMDARs at the PF-PC synapse would affect LTP induction (Fig. 3*A*). Given that only Tg2 showed significantly more NMDA-mediated currents at their PF-PC synapses than controls, we focused our plasticity experiments on Tg2 and their wild-type littermates. Indeed, whereas the controls showed normal potentiation (pre- vs post-tetanus: *p* < 0.001, linear mixed model; Materials and Methods), the amplitude of the EPSCs of the Tg2 mice after LTP induction was significantly lower from the ones recorded in control cells (Tg2 vs Ctrls *p* = 0.034, repeated measures ANOVA). In contrast, LTD at the PF-PC synapse (Ctrls, pre- vs post-tetanus: *p* < 0.05, linear mixed model; Materials and Methods) was not affected (Tg2 vs Ctrls, *p* = 0.63, repeated measures ANOVA; Fig. 3*B*). To unequivocally link the phenotype in LTP induction to the activation of NMDARs we repeated the experiment in presence of NMDAR antagonist D-AP5 (Fig. 3*C*). Blocking NMDA currents minimized the effects on the induction of LTP (pre- vs post-tetanus for Ctrls, *p* < 0.001, linear mixed model; Materials and Methods), ablating the difference between genotypes (*p* = 0.80, repeated measures ANOVA).

Impairment in PF-PC LTP induction often co-occurs with long-term deficits in intrinsic plasticity (IP) (Belmeguenai et al., 2010; Schonewille et al., 2010; Peter et al., 2016). Instead, in PCs of Tg2 mice the overall IP was enhanced shortly after induction (p = 0.036 for the first 10 min after induction, 0.095 for the entire period, repeated measures ANOVA; Fig. 3*D*). We conclude that overexpression of NMDAR’s in PCs selectively prevents the induction of LTP at the PF-PC synapse, and that this deficit might be partly compensated for by a modest early increase in IP.

### Normal motor behavior but impairment in motor learning

**Figure 4.**
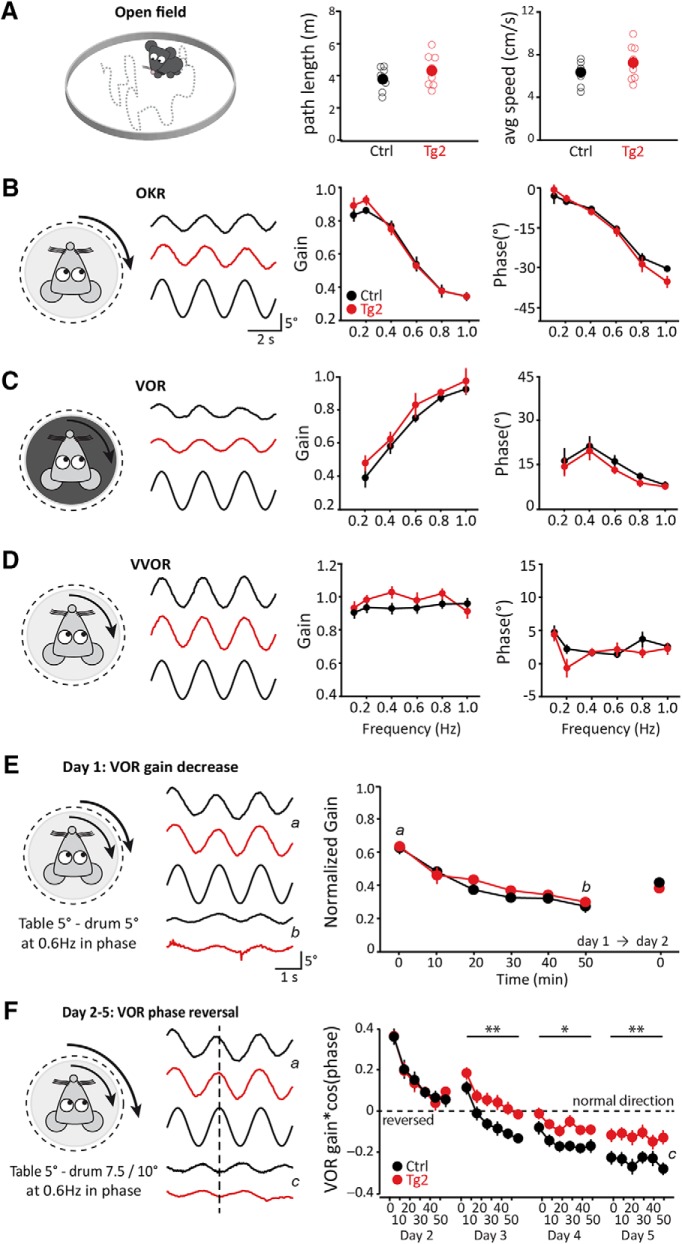
Motor performance is normal, but motor learning is impaired in transgenic mice. ***A***, Distance traveled and average speed in the open field for transgenic (Tg2, red, *n* = 8) and control (Ctrl, black, *n* = 7) mice. ***B–D***, Baseline compensatory eye movements (examples traces for 0.4 Hz, middle left) quantified by gain (middle right) and phase (right) for Tg2 mice (red, *n* = 9) and control (Ctrl, black, *n* = 10) mice: (***B***) OKR; (***C***) VOR (in the dark); and (***D***) VVOR (in the light), schematized on the left of each respective panel. ***E***, left, Representation of gain-decrease training paradigm (day 1: 5 × 10 min sinusoidal, in-phase drum and table rotation at 0.6 Hz, both with an amplitude of 5°; day 2: VOR gain measurement at 0.6 Hz). Middle, Example traces of before (time point, t = 0, indicated by *a*) and after (*t* = 50 min, *b*) adaptation. Right, Normalized gain for VOR recorded with 10-min intervals during 50-min training session for six-week-old Tg2 mice (red, *n* = 8) and control (Ctrl, black, *n* = 11) mice on day 1 and a single measurement at day 2. ***F***, Similar to ***E***; following the gain-decrease protocol, for four consecutive days the six-week-old transgenic and control mice were subjected to the phase reversal protocol (5 × 10 min sinusoidal in-phase drum and table rotation at 0.6 Hz, but with drum amplitudes of 7.5° on day 2 and 10° on days 3–5, while the table amplitude was 5°). VOR responses (middle: example traces, *a* same as ***E***, *c*: *t* = 50 min on day 4) are depicted as gain of eye movement multiplied by the cosine of its phase, gain*cos(phase). Negative values here indicate a phase larger than 90° and the (theoretical) goal of the training is a value of -1. Empty circles represent individual data points, full circles are mean ± SEM; *p* values are indicated in the main text; asterisks indicate significant difference.

LTP at the PF-PC synapse probably does not affect baseline motor performance, but it may well contribute to vestibulo-cerebellar motor learning (De Zeeuw et al., 2011; Gao et al., 2012; Ito, 2002). At the same time, it should be noted that short-lasting enhanced IP, as observed in the Tg2 mutant, might partly compensate for a deficit in LTP induction (De Zeeuw et al., 2011; Gao et al., 2012). To evaluate the behavioral consequences of overexpression of NR2B in the PCs of the Tg2 mice we subjected them to both motor performance and learning tests. First, we confirmed that the general motor behavior of Tg2 animals was comparable to that of their control littermates in an open field test; neither path length nor average speed was affected (*p* = 0.28 and *p* = 0.29, respectively; Fig. 4*A*). In addition, we evaluated oculomotor activity, which is particularly sensitive to cerebellar deficits (Ito, 2002; Boyden et al., 2006; Schonewille et al., 2010). Both gain and phase values during the OKR as well as during the VOR in the dark and VVOR elicited by sinusoidal stimulation at different frequencies (0.1–1.0 Hz) with a fixed amplitude (5°) did not differ significantly between genotypes (all *p* values > 0.13, repeated measures ANOVA; Fig. 4*B–D*).

Next, we subjected the Tg2 animals to the type of VOR training that is most sensitive, i.e., phase reversal learning, which is aimed at gradually converting the direction of the eye movements over several days of in-phase vestibular and visual stimulation (Badura et al., 2016). During the first session, which entails standard gain-decrease training evoked by rotating drum and table in phase at 5° at 0.6 Hz, the amplitude of the VOR in Tg2 mice decreased to similar levels as that in control mice (*p* = 0.33, repeated measures ANOVA; Fig. 4*E*).

Moreover, when the animals were tested again after spending 23 h in the dark, the consolidation of the change in VOR gain was not different among genotypes (*p* = 0.21). However, when we subjected animals to phase reversal training for four consecutive days, the Tg2 mice performed worse than controls in that their adaptation was delayed. The training aims to reverse the direction of the VOR, resulting in a negative gain*cos(phase) value (Fig. 4*F*) and this adaptation is impaired in Tg2 mouse from day 3 onwards (day 3, 4, and 5: *p =* 0.005, *p =* 0.026, and *p =* 0.001, respectively, repeated measures ANOVA). Together, these data show that NR2B transgenic mice have an unaffected baseline motor performance and that their learning capabilities are slightly, but significantly, affected.

## Discussion

To shed light on the surprisingly low expression level in PCs of the main coincidence detector in the brain, the NMDAR, we generated a transgenic mouse line that overexpresses NMDARs at one of the most studied and phylogenetically oldest sites of plasticity in the brain, the PF-PC synapse. The L7-NR2B+ Tg2 mice, which expresses functional NMDAR-mediated currents not only at the CF-PC synapse but also at the PF-PC synapse, develop normally and have no morphologic abnormalities or impaired motor performance. Interestingly, the NMDAR-mediated currents abolishes the ability for LTP induction at the PF-PC synapse and affects a demanding form of cerebellar-dependent motor learning, VOR phase-reversal learning.

As previously shown in an *in vitro* essay (Kakegawa et al., 2003), our data imply that NR1 subunits are sufficiently expressed in PCs to aggregate with the exogenously expressed NR2 subunits to form functional heterotetramers. As low expression levels and suboptimal antibody quality prohibit determining the subcellular localization of the NR2B subunit in our experiments, we cannot exclude the possibility of extra-synaptic receptors being also present in these transgenic lines. The normal absence of NMDARs at PF input sites under physiological circumstances is therefore not caused by the scarceness of NR1 subunits, but by the limited and finely regulated NR2 expression and selective intracellular transport machinery (Piochon et al., 2007; Renzi et al., 2007), which may in part depend on the UTR sites of the subunits (Di Liegro et al., 2014; Wood et al., 1996). Moreover, it also appears that NMDARs are not absent from the PF-PC synapse in controls to prevent excitotoxicity (Slemmer et al., 2005), as we found no sign of PC death in our transgenic mouse lines. Still, the amount of NMDAR-mediated current was not massive, and we cannot exclude the possibility that higher expression levels could potentially trigger PC apoptosis.

The main consequence of the genetically induced presence of NMDARs at the PF-PC synapse is that it renders the synapse incapable of potentiation. In contrast to other well-studied excitatory synapses, e.g., the well-characterized hippocampal CA3 to CA1 synapse or the synapses formed by the cerebellar mossy fibers with granule cells and cerebellar nuclei neurons (Bliss and Lomo, 1973; D'Angelo et al., 1999; Pugh and Raman, 2006), NMDARs at the PF-PC synapse are not only dispensable for LTP induction, they are in fact effectively blocking it if present at the postsynaptic site. The plasticity induction rule in PCs is, in terms of calcium dependence, reversed compared to the traditional BCM-rule in pyramidal cells (Coesmans et al., 2004; Bienenstock et al., 1982); hence, LTP induction is only possible when Ca^2+^ concentrations are relatively low. In fact, in wild-type mice the presence or absence of CF-triggered Ca^2+^ level increase determines the occurrence of LTD or LTP, respectively, and this effect is independent of the change in Ca^2+^ levels evoked by PF stimulation (Piochon et al., 2016). It is therefore tempting to hypothesize that in our transgenic mice the presence of Ca^2+^-permeable NMDARs increases the Ca^2+^ concentration to a level that is too high to allow LTP, while it leaves LTD induction unaffected. Conversely, but in line with this concept, blocking Ca^2+^-permeable NMDARs in mature mice reduces the dendritic Ca^2+^ influx during a complex spike to a level that is too low for LTD, while it leaves LTP unaffected (Piochon et al., 2010). Such a mechanism, which will have to be looked at in future studies, may also explain why IP was slightly increased in the Tg2 mice, as this form of plasticity is largely regulated by the activity of SK2 channels, which is also Ca^2+^ dependent (Maher and Westbrook, 2005; Hosy et al., 2011). In this regard, the L7-NR2B+ Tg2 mice diverge from other LTP-deficient mutants in which the calcium dynamics are not directly affected. PC-specific mutants, such as the L7-PP2B-, L7-Shank2-, and L7-GluR3-mutants (Schonewille et al., 2010; Peter et al., 2016; Gutierrez-Castellanos et al., 2017), all do not only show a blockage of LTP induction, but they also show a profound reduction in IP, rather than an enhancement as found here in the NR2B-Tg2 mice. The combination of deficits in both LTP induction and IP leads to a more severe behavioral phenotype in that the VOR phase reversal adaptation is, unlike that of the Tg2 mice, virtually completely abolished (Schonewille et al., 2010; Peter et al., 2016; Gutierrez-Castellanos et al., 2017). Thus, the relatively mild behavioral phenotype of the Tg2 mice may be explained by an intact, if not elevated, level of IP, which might also at least partly result from an elevated Ca^2+^.

Our results fit with the interpretation that different types of plasticity in PCs, and in other cerebellar neurons, synergistically interact to ensure optimal learning (Gao et al., 2012; Galliano and De Zeeuw, 2014). According to this theory, mutations that impair several plasticity mechanisms or network elements simultaneously typically affect more basic types of cerebellar-dependent learning and, if they do so, they affect them more severely (Ichise et al., 2000; Wulff et al., 2009; Schonewille et al., 2010; Galliano et al., 2013). In contrast, when a single mechanism at a single type of synapse in the circuit is impaired, other mechanisms may compensate (Gao et al., 2012) and the ability to perform and learn motor tasks may be maintained to a larger extent (this manuscript; Schonewille et al., 2011).

## Synthesis

Reviewing Editor: Christian Hansel, University of Chicago Decisions are customarily a result of the Reviewing Editor and the peer reviewers coming together and discussing their recommendations until a consensus is reached. When revisions are invited, a fact-based synthesis statement explaining their decision and outlining what is needed to prepare a revision will be listed below. The following reviewer(s) agreed to reveal their identity: Claire Piochon

In this study the authors generated transgenic mice overexpressing NR2B subunits in cerebellar Purkinje cells to examine the effect of ectopic expression of NMDA receptors (NMDAR) at parallel fiber (PF) to Purkinje cell (PC) synapses where NMDA receptors are not expressed normally. While it has been shown previously that virally expressed NR2B subunits can form functional NMDAR at PF synapses, it remained unknown whether these ectopic receptors would impact Purkinje cell activity and motor learning. Very interestingly the authors show that in the transgenic mice larger NMDAR-mediated currents block LTP induction at PF-PC synapses, which is associated with the impairment of the adaptation of the Vestibulo-Ocular Reflex (VOR). Combining histology, electrophysiology and behavior, this study presents a thorough characterization of the transgenic mice. Overall the results shed light on a surprising specificity of the PF-PC synapse, a phylogenetically conserved site of plasticity that is crucial for cerebellar physiology, and a model in synaptic plasticity studies. By providing advances in the field I believe this interesting article will be suitable for publication in eNeuro but some revisions would improve the impact of the paper. The topic and results are interesting, if the results support conclusions. However, I have several concerns, in particular, about the quality of data presented as described below.

The problematic of the study stated in the abstract consists in addressing why NMDAR are absent from PF-PC synapses. While the results suggest that the presence of NMDAR would disturb the tight control of calcium level required for the different types of plasticities occurring at PF synapses, the results do not explain what can cause the selective absence of these receptors from PF synapses. NR3B subunits are expressed in mature PC in rats (Wee et al. 2008, DOI: 10.1002/cne.21747). Since these subunits can form heteromers with NR1 and NR2B subunits and reduce NMDAR calcium permeability and sensitivity to Mg2+, it could be interesting to look at the NR3B labeling in the transgenic mice.

According to Renzi et al. (2007), ifenprodil partly inhibits somatic NMDAR in somatic patches but not in the synaptic currents suggesting that NR2B subunits remain extra synaptic in PC while NR2A are more probably synaptic. Is this localization modified in the transgenic mice? The histology section would benefit from higher magnification pictures of NR2B staining in PC in confocal microscopy.

By overexpressing NMDA receptors in PCs, the study teaches us more about the calcium dependency of PF-PC plasticities than on the characteristic absence of NMDAR from PF synapses. Thus to highlight the necessity of a tight control of calcium level in these synapses, first the authors should provide an updated discussion, incl. Piochon et al. PNAS 2016. Second, it would be interesting to test if NMDA overexpression also impairs LTP induced by protocol involving high frequency stimulation of PF (Piochon et al. 2010, J. Neurosci.).

Further major concerns:

1, Apparently NR2B protein was expressed more in Tg1 than in Tg2 according to Western blot results presented in Fig 1B. First, quantification of NR2B amount should be presented. If NR2B is expressed more in Tg1, why NMDA current at synapses on Purkinje neurons was larger in Tg2 than in Tg1 (Fig 2A, B)? Explanation is required.

2, Clearer and more magnified photographs showing Purkinje cell dendrites such as shown in Figure 4 of Belmeguenai et al., (2010, JNS) should be presented in Figure 1D in order to demonstrate normal morphology of Purkinje cells in transgenic mice. Alternatively, authors could present single Purkinje cell morphology by injecting fluorescent dye or HRP.

3, Why was the time course of NMDA current at PF-PC synapses much faster in Tg2 than in Tg1 (Figure 2B green traces, Tg1 and Tg2)? Why is the blue trace missing in Fig2B Tg2?

4, I am not convinced that LTD was induced in the experiments presented in Fig 3B. The amplitudes of EPSCs were close to the basal level at 24-28 minutes, and then decreased. These time courses are not natural, and I suspect that the decrease of EPSP by tetanus might not be statistically significant at 24-28 minutes in both control and transgenic mice. Statistical comparison between pre VS post seems to have been performed only at 20 min after tetanus (methods P9). Data from later time points such as at 25 or 30 min should be used.

5, I am also not convinced that LTP was induced in the experiments with APV presented in Figure 3C. Were they statistically different at 25 or 30 min? Compared with the results without APV (Fig 3A Ctrl) LTP looks suppressed. Isn't the difference statistically significant? If LTP was suppressed by APV, it is inconsistent with the results reported by Piochon et al., (2010, JNS, Figure 2B). What was the cause of difference? Was LTP reported here (Figure 1A) postsynaptic one but not presynaptic LTP? The authors can examine whether pre- or postsynaptic LTP occurs by applying paired pulse stimulation.

6, Representative EPSC traces should be presented in Figure 3A-C.

7, I do not agree with the authors' claim that “in PCs of Tg2 mice the overall intrinsic plasticity tended to be enhanced” (Fig 3D, line 16 P16). Alternative interpretation is that some short-lasting suppressive phenomenon of intrinsic excitability was induced in control mice and this effect was suppressed in Tg2 mice. The time course of intrinsic excitability change in control mice (Fig 3D) look different from that of the previously reported potentiating plasticity of intrinsic excitability (Belmeguenai et al., 2010 JNS). What was the cause of difference? Intrinsic plasticity can be also induced by depolarization of PC (Belmeguenai et al., 2010 JNS). Is depolarization induced intrinsic plasticity affected by overexpression of NR2B? Voltage traces showing action potential firing patterns before and after the tetanus should be presented.

8, Eye position or velocity traces should be presented in Figure 4B-D, F.

Minor comments

1, P 8, line 5-6, “glycine (10 mM), NBQX (12.5 mM) D-AP5 (10 mM)”, ‘mM’ might be ‘microM’?

2, P11, line 4. The maker name and the product number of infrared emitter should be provided.

3, How did the author determine data structure (Table 1). Statistical test to confirm normal distribution should be described.

4, P29 line 1, ‘significant’ might be ‘significantly different’.

5, in the methods, use of bicuculine or picrotoxin in the experiments is not clearly reported.

6, Page 15, last paragraph, please precise that PF-LTP has been shown to not depend on post-synaptic NMDAR (Piochon et al., 2010; Wang et al., 2014 J.Neurosc).
